# (Re)Defining the Proline-Rich Antimicrobial Peptide Family and the Identification of Putative New Members

**DOI:** 10.3389/fchem.2020.607769

**Published:** 2020-12-01

**Authors:** Nicholas G. Welch, Wenyi Li, Mohammed Akhter Hossain, Frances Separovic, Neil M. O'Brien-Simpson, John D. Wade

**Affiliations:** ^1^The Florey Institute of Neuroscience and Mental Health, University of Melbourne, Melbourne, VIC, Australia; ^2^School of Chemistry, University of Melbourne, Melbourne, VIC, Australia; ^3^Centre for Oral Health Research, Melbourne Dental School, University of Melbourne, Melbourne, VIC, Australia; ^4^Bio21 Institute, University of Melbourne, Melbourne, VIC, Australia

**Keywords:** 70S ribosome, AMPs, antimicrobial peptides, DnaK, host defense peptides, PrAMP, proline-rich antimicrobial peptide

## Abstract

As we rapidly approach a post-antibiotic era in which multi-drug resistant bacteria are ever-pervasive, antimicrobial peptides (AMPs) represent a promising class of compounds to help address this global issue. AMPs are best-known for their membrane-disruptive mode of action leading to bacteria cell lysis and death. However, many AMPs are also known to be non-lytic and have intracellular modes of action. Proline-rich AMPs (PrAMPs) are one such class, that are generally membrane permeable and inhibit protein synthesis leading to a bactericidal outcome. PrAMPs are highly effective against Gram-negative bacteria and yet show very low toxicity against eukaryotic cells. Here, we review both the PrAMP family and the past and current definitions for this class of peptides. Computational analysis of known AMPs within the DRAMP database (http://dramp.cpu-bioinfor.org/) and assessment of their PrAMP-like properties have led us to develop a revised definition of the PrAMP class. As a result, we subsequently identified a number of unknown and unclassified peptides containing motifs of striking similarity to known PrAMP-based DnaK inhibitors and propose a series of new sequences for experimental evaluation and subsequent addition to the PrAMP family.

## Introduction

Antimicrobial peptides (AMPs) are a well-known class of naturally occurring compounds with potent bactericidal activity. They typically exert their antimicrobial efficacy via membrane disruption leading to lysis (Sheard et al., 2019). However, a subclass known as proline-rich AMPs (PrAMPs) are membrane permeable and non-lytic (Li et al., 2014). Instead, PrAMPs generally act on intracellular targets and inhibit protein synthesis leading to bacteria death (Scocchi et al., 2011). The major transporters for PrAMP uptake are Gram-negative inner membrane proteins SbmA and YgdD, though MdtM also plays a role particularly at higher PrAMP concentrations (Krizsan et al., 2015; Paulsen et al., 2016; Graf and Wilson, 2019).

PrAMPs were first discovered in 1989 with the identification of apidaecin in the lymph fluid of the honey bee *Apis mellifera* (Casteels et al., 1989). PrAMPs were subsequently identified in cow neutrophils [bactenecins 5 and 7 (Gennaro et al., 1989)], again in bees with the discovery of abaecin (Casteels et al., 1990), and in pig intestine (PR-39–Agerberth et al., 1991). Drosocin from fruit flies was identified in 1993 (Bulet et al., 1993) and pyrrhocoricin was discovered in sap sucking bugs in 1994 (Cociancich et al., 1994). A PrAMP family isolated from *Palomena prasina*, coined the metalnikowins, were discovered in 1995–1996 (Chernysh et al., 1996). The latter is not to be confused with the metchnikowin peptides also discovered in 1995 in Drosophilia (Levashina et al., 1995). Shrimp penaeidins were identified in 1997 (Destoumieux et al., 1997) and ant formaecins in 1998 (Mackintosh et al., 1998). Oncopeltus antibacterial peptide-4 (named as oncocin) was discovered in 2001 from the milkweed bug (Schneider and Dorn, 2001). Heliocin from the tobacco budworm moth (Heliothis virescens) was submitted to UniProt in 2002 (NCBI#P83427). Ovine bactenecin OaBac6 was discovered in 2005 (Huttner et al., 1998). Arasin was discovered in crabs in 2008 (Stensvag et al., 2008) and the abaecin-like peptide PP30 was identified in wasps in 2010 (Shen et al., 2010). From the bean bug (*Riptortus pedestris*), riptocin was discovered in 2015 (Kim et al., 2015) as was BnPRP1 (Cao et al., 2015), the first proline-rich peptide discovered in plants (*Brassica napus*). In 2018, the first PrAMPs discovered in dolphins were Tur1A and Tur1B (Mardirossian et al., 2018).

Since their discovery, a number of strategies have been adopted to develop improved PrAMP analogs including the use of consensus sequence analysis, resulting in a *de novo* A3-APO (All Peptide Optimized) peptide (Otvos et al., 2005), and of brute force large library analogs leading to bactenecin 5 derivatives with improved spectrum of activity being developed some 30 years after the discovery of the original bactenecin 5 (Mardirossian et al., 2019). Additionally, PrAMPs are of great interest as potential vectors for drug delivery, with pyrrhocoricin, bactenecin 7, and PR-39 each being reported to be cell penetrating peptides and oncocin, apidaecin and drosocin also able to cross the blood-brain barrier (Li et al., 2014). More recently, the introduction of proline residues has been used to improve the therapeutic index of AMPs (Azuma et al., 2020). Hence, understanding the primary structural features of naturally occurring PrAMPs has important relevance to future therapeutic development.

**Figure 1:**
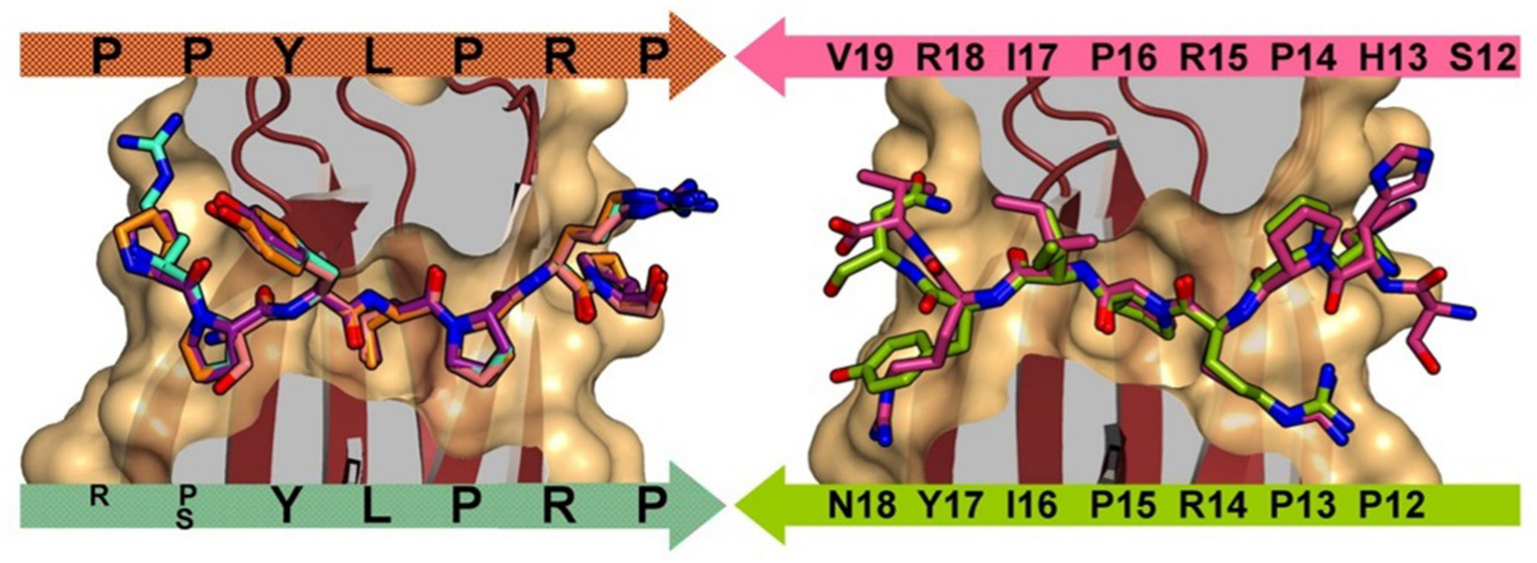
Left: DnaK forward binding mode shown by superposition of A3-APO (1–20) (cyan), Onc72 (orange), PR-39 (1–15) (purple), and pyrrhocoricin (1–20) (salmon). Right: DnaK reverse peptide binding mode shown by superposition of pyrrhocoricin (12–20) (green) and drosocin (12–19) (purple). Reproduced from Zahn et al. (2013) with permission.

Otvos et al. first identified that the PrAMPs pyrrhocoricin, drosocin, and apidaecin interacted with the heat shock protein 70 (Hsp70, also known as DnaK in bacteria) (Otvos et al., 2000) and later found that this PrAMP family also inhibited chaperone-assisted protein folding (Otvos et al., 2001). For a number of PrAMPs, binding to DnaK has been shown to proceed through the substrate cleft and, based on the peptide orientation within the cleft, can be either in the forward or reverse binding mode (Figure 1) (Zahn et al., 2013). Some PrAMPs, such as pyrrhocoricin, demonstrate both forward and reverse modes through distinct motifs. Although PrAMPs bind to DnaK, antibacterial testing using DnaK deletion strains of *Escherichia coli* found only modest variations in minimum inhibitory concentration (MIC) and minimum bactericidal concentration (MBC) compared to that induced in the wild type *E. coli* (Scocchi et al., 2009).

**Figure 2:**
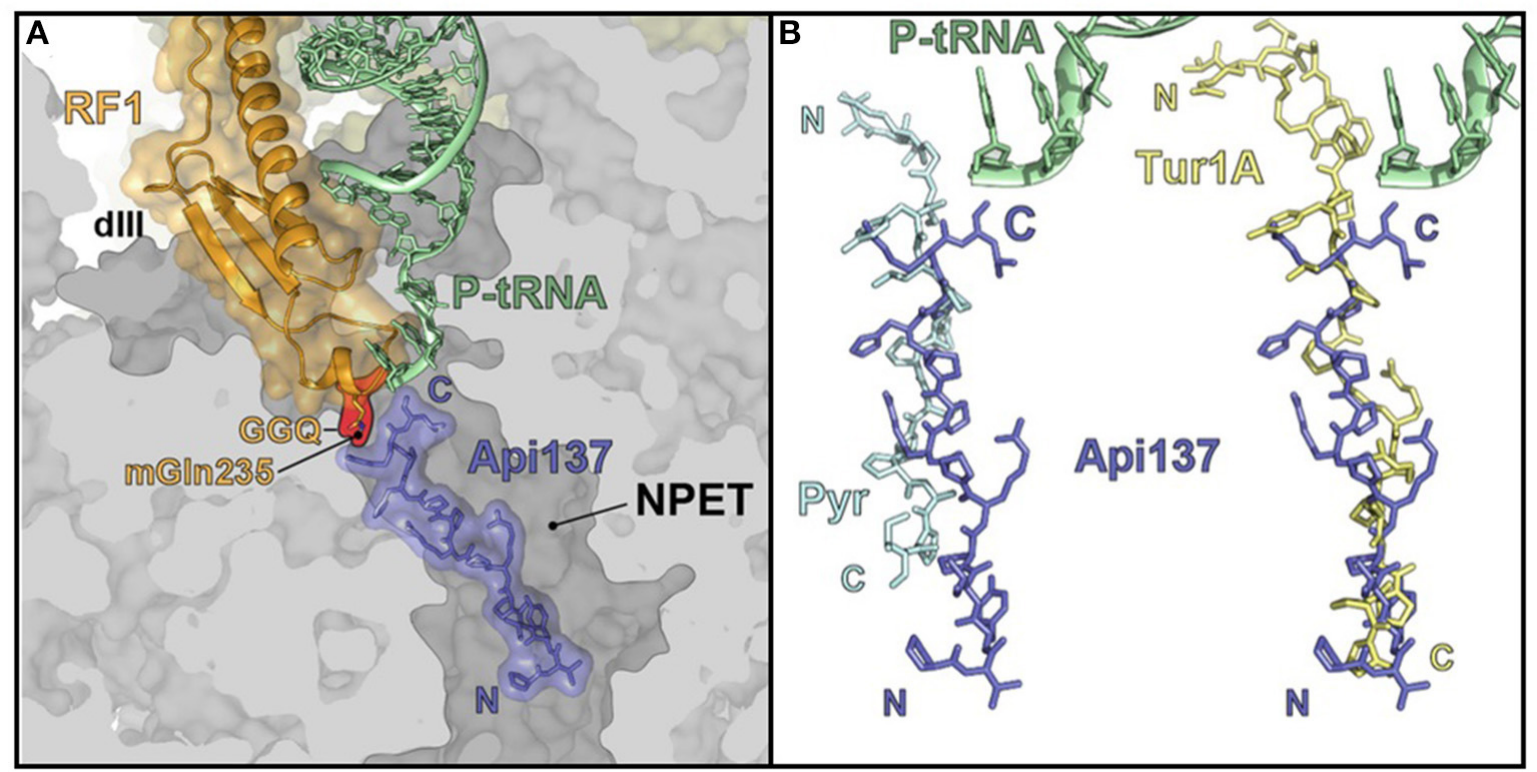
PrAMP binding at the 70S ribosome. **(A)** Api137 locates within the ribosomal large subunit polypeptide exit tunnel (NPET) in the presence of peptidyl-transfer RNA (P-tRNA) and release factor 1 (RF1). **(B)** Api137 binds in an inverted orientation relative to pyrrhocoricin (Pyr) and Tur1A with the N-termini and C-termini marked by N and C, respectively. Reproduced from Graf and Wilson (2019) with permission.

Consequently, Hoffmann et al. proposed a new mechanism whereby PrAMPs inhibited protein translation acting through the 70S ribosome (Krizsan et al., 2014). The PrAMPs were divided into two classes based on the conformation in which they bind to the ribosome (Figure 2A). Class I PrAMPs act by preventing the first translation elongation step and bind at the polypeptide exit tunnel (NPET) in an extended conformation and in an inverted orientation relative to the nascent chain (Graf and Wilson, 2019). Members of Class I include an oncocin derivative Onc112, bactenecin 7, metalnikowin and pyrrhocoricin. In each of these cases, a characteristic motif, Pro-Arg-Pro (PRP), is located in the same position and with the same conformation in the binding site (Graf and Wilson, 2019). Currently, the Class II PrAMPs only include apidaecin 1b and Api137 (derived from apidaecin 1b) and bind in a similar orientation to the nascent chain and predominately act as translation termination inhibitors. The difference in binding orientation between the Class II PrAMP Api137 and Class I PrAMPs pyrrhocoricin (Pyr) and Tur1A is shown in Figure 2B.

A prevailing question for PrAMPs is whether they are all inhibitors of either or both DnaK and the 70S ribosome and, furthermore, what, if any, are the defining structural features of peptide members of the PrAMP family. As yet, no concrete definition has been proposed for PrAMPs although the literature offers a number of broad definitions that can be individually and collectively analyzed and distilled to provide a refined definition that enables the identification of potential new members of the family known as PrAMPs.

## Historical PrAMP Definitions Applied to Known Amps

**Figure 3:**
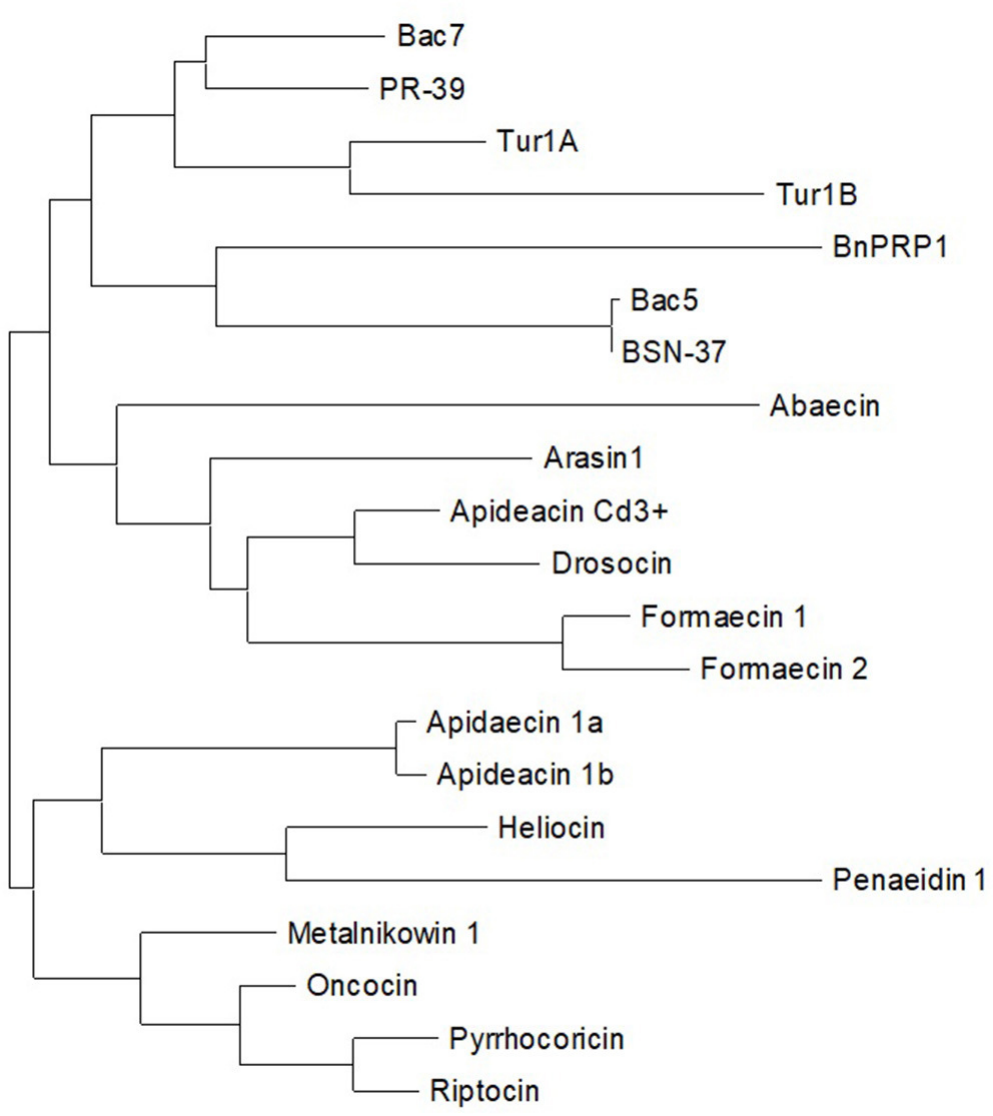
Phylogenetic tree of naturally occurring PrAMPs identified from the literature.

To develop an improved, updated definition of PrAMPs, we first reviewed the literature for past and current definitions and then analyzed known members of the family to gain further insight as to where the boundaries of the class might lie. Beginning with an overview of known PrAMPs from recent reviews and literature, we then sequentially assessed how well literature definitions of PrAMP characteristics fit these known peptides. Additionally, we also considered these constraints as applied to the DRAMP database (http://dramp.cpu-bioinfor.org/) and explored particular examples that fit the current PrAMP literature definitions. Table 1 summarizes several PrAMPs identified from the literature and highlights key characteristics of these peptides. The inferred evolutionary analysis of these peptides is shown in a phylogenetic tree in Figure 3.

**Table 1:** Naturally occurring PrAMPs identified from the literature.

**Name**	**Sequence**	**Length**	**Pro (%)**	**Arg (%)**	**Net charge**	**PRP motifs**
Abaecin (*Apis mellifera*)	YVPLPNVPQPGRRPFPTFPGQGPFNPKIKWPQ	32	31%	6%	+4	0
Apidaecin 1a	GNNRPVYIPQ**PRP**PHPRI	18	33%	17%	+3	1
Apidaecin 1b	GNNRPVYIPQ**PRP**PHPRL	18	33%	17%	+3	1
Apidaecin Cd3+	GKPSK**PRP**APIK**PRP**PHPRL	20	40%	15%	+6	2
Arasin1	SRWPSPGR**PRP**FPGRPKPIFR**PRP**C	25	36%	24%	+7	2
Bac5(1–23)	RFRPPIRRPPIRPPFYPPFRPPI	23	43%	26%	+6	0
Bac7(1–35)	RRIR**PRP**PRL**PRPRPRP**LPF**PRP**G**PRP**I**PRP**LPFP	35	46%	31%	+11	6^#^
BnPRP1	PPTQNPSMAPPTQNPYGQPMTPPTQNPYGQPMAPP	35	37%	0%	0	0
BSN-37	FRPPIRRPPIRPPFYPPFRPPIRPPIFPPIRPPFRPP	37	49%	22%	+8	0
Drosocin	GK**PRP**YS**PRP**TSH**PRP**IRV	19	32%	21%	+5	3
Formaecin 1	GRPNPVNNKPTPYPHL	16	31%	6%	+2	0
Formaecin 2	GRRNPNNKPTPHPRL	15	27%	20%	+4	0
Heliocin	RFIHPTYRPPPQPRRPVIMRA	21	29%	24%	+5	0
Metalnikowin 1	VDKPDYR**PRPRP**PNM	15	33%	20%	+2	1^#^
Oncocin	VDKPPYL**PRPRP**PRRIYNR	19	32%	26%	+5	1^#^
Penaeidin-1	YRGGYTGPI**PRP**PPIGRPPLRLVVCACYRLSVSDARNCCIKFGSCCHLVK	50	14%	12%	+7	1
PR-39	RRR**PRP**PYL**PRPRP**PPFFPPRLPPRIPPGFPPRFPPRFP	39	49%	26%	+10	2^#^
Pyrrhocoricin	VDKGSYL**PRP**TP**PRP**IYNRN	20	25%	15%	+3	2
Riptocin	VDKGGYL**PRP**TP**PRP**VYRS	19	26%	16%	+3	2
Tur1A	RRIRFRPPYL**PRP**GRRPRFPPPFPIPRIPRIP	32	38%	31%	+10	1
Tur1B	RRIPFWPPNWPGPWLPPWSPPDFRIPRILRKR	32	31%	19%	+6	0

*PRP motifs are bolded. The hash (^#^) indicates overlapping PRP motifs*.

### Proline Content and the PRP Motif

Several qualifiers have been stated to describe PrAMPs in terms of their proline content and/or having the defined motif Pro-Arg-Pro (PRP) (Mishra et al., 2018). Regarding the total proline content within a PrAMP sequence, a number of studies have proposed differing percentages of Pro content e.g., >25% proline (Gillespie et al., 1997), about 30% proline content (Mishra et al., 2018), and a “high” proline content (Graf and Wilson, 2019). Examining the reported PrAMPs in Table 1 it is observed that these definitions fit the known sequences quite well. The proline content range is 14–49% with a mean of 34%. Only the shrimp penaeidin-1 peptide at 14% proline is below the >25% definition, whereas four of the peptides (Bac5(1-23), Bac7(1-35), BSN-37, and PR-39) are over 40% proline. Based on the consensus of these sequences, we propose that the minimum proline content be 25%, ensuring that the proline content comprises a significant portion of the overall peptide sequence. Using this minimal proline content definition, penaeidin-1 (and the penaeidin family) would no longer be considered to be PrAMPs. However, it should be noted that the 25% proline content is an arbitary threshold for members of the PrAMP family and truncated analogs may warrant PrAMP membership despite the parent peptide being below the threshold. In the case of the penaeidin-1, the proline-rich domain (PRD), preceeding the cysteine-rich domain (CRD) in most penaeidins (Tassanakajon et al., 2011), contains 7 prolines in a total of 24 residues (29%); thus meeting the minimum proline content proposed here. Interestingly, in the related penaedin, the PRD of penaeidin4-1 (but not penaeidin3-4) captured the antimicrobial properties of the full-length sequence against multiple strains of Gram-positive bacteria though failed to inhibit Gram-negative *E. coli* growth (Cuthbertson et al., 2006). In summary, while the PRD meets the minimal proline content of PrAMP and that the PRD may be the active portion of the penaeidin-1 peptide, to the best of our knowledge, no direct evidence of it's bactericidal activity is provided in the literature. Thus, antimicrobial assays using synthetic peptides would be required to confirm its antimicrobial activity.

Analysis of the DRAMP database of 4,745 peptides revealed a proline content range of 0–53% with 2,837 sequences (i.e., 60% of the database) >0% proline with a mean of 7%. Regarding the proposed constraint of 25% or greater, there are 98 sequences meeting this criterion comprising 2.1% of the DRAMP database. This is a significant portion of known AMPs containing a relatively high content of proline and forms a key basis for our identification of new members of the PrAMP family.

Literature definitions also highlight that PrAMPs may “contain one or several Pro-Arg-Pro motifs” (Mishra et al., 2018) with this motif implicated in DnaK binding. Considering the PrAMPs listed in Table 1, they contain up to 6 PRP motifs in a single sequence. However, 8 of the 21 sequences in the table, including Bac5(1–23), abaecin, heliocin, and formaecin, do not contain the PRP motif which indicates that this motif alone is insufficient to describe all members of the PrAMP family. A recent structural study on the PrAMP binding domain of DnaK demonstrated that PRP either led or trailed the “active” part of the sequence that often contained a leucine and tyrosine (e.g., YLPRP) (Zahn et al., 2013). Additionally, regarding binding to the 70S ribosome, the central arginine of the PRP motif plays an important role in the binding of insect PrAMPs pyrrhocoricin, metalnikowin and Onc112, and also mammalian PrAMPs bactenecin 7 and Tur1A, participating in hydrogen bonding with the 23S nucleotide U2484 and stacking with C2610 (Graf and Wilson, 2019).

Interestingly, substitution of the central arginine of the PRP motif with a lysine (PKP) resulted in a 2-fold reduction in antibacterial efficacy with apidaecin and drosocin (Lele et al., 2013). Whereas, for pyrrhocoricin substituted with L7K and R14K (VDKGSY**K**PRPTPP**K**PIYNRN, i.e., one PRP modified, one unmodified, and an increase in net charge with L7K), an increase in antibacterial efficacy by 2 to 5-fold was observed (Lai et al., 2019). However, using inner membrane transport protein knockout *E. coli* strains (ΔSbma and ΔYgdD), the authors demonstrated that this increase in efficacy may also be the result of increased membrane binding and internalization.

Whilst the PRP motif might be a strong indicator that a peptide is a likely DnaK binder, the presence of the motif *alone* is insufficient to predict members of the PrAMP family as many other residues are known to participate in the binding of PrAMPs to either DnaK or the ribosome (Graf and Wilson, 2019). For example, the synthetic short peptides NRLLLTG and ELPPVKI are binders of DnaK with micromolar K_d_ (Zahn et al., 2013), however are not known to be membrane permeable.

### Charge

PrAMPs have been described as commonly being cationic (Graf and Wilson, 2019) whereby positively charged residues enhance interaction with the bacterial cell wall or membrane. However, this is not to be confused with typical membrane-lytic antimicrobial modes of action since PrAMPs are largely non-lytic (Li et al., 2014). The net charge range for the PrAMPs in Table 1 is 0 to +11 with a mean of +5. With the exception of BnPRP1 [thus far the only PrAMP to be isolated from plants, (Cao et al., 2015)], all other sequences are cationic which indicates that net charge might be a suitable definition qualifier for the PrAMP family. Additionally, given that the mean charge is +5 for these PrAMPs it may be that there exists a minimum charge threshold for membrane interaction and permeation.

Commonly for these PrAMPs this cationicity is contributed to by at least three arginine residues. One reference has highlighted the necessity of a “high” arginine content, alongside proline content, as a defining property of PrAMPs (Graf and Wilson, 2019). For these naturally occurring PrAMPs, the arginine content ranges from 0 to 31% with a mean of 19%. Four sequences have (arbitrarily) <15% arginine: BnPRP1 (0%), formaecin 1 and abaecin (6%), and penaeidin-1 (12%). At this point, arginine does not appear to be a significant factor to describe PrAMPs, as such we propose that arginine content is not a suitable qualifier to include or omit members from the PrAMP family. Rather, it may be that the main contribution from most arginine residues in PrAMPs might be their charge, and perhaps lysine or histidine residues are also suitable substitutions to preserve membrane interactions and DnaK or ribosome inhibition. Under this charge definition, again, BnPRP1 would not be considered a member of the PrAMP family.

AMPs identified from the DRAMP database had a diverse net charge ranging from −20 to +30. Of those peptides with a positive net charge (cationic), 3,815 sequences (80.4%) were identified. This is unsurprising as peptides with a lytic mechanism of action commonly have a net positive charge to promote membrane embedding. Cationic nature is undoubtedly an important predictor for PrAMPs. However, given the prevalence of cationic AMPs, other features in combination with charge are clearly required for differentiation of PrAMPs from general AMPs.

### Length

One reference describes that PrAMPs commonly have a sequence length of 18–34 amino acid residues (Gillespie et al., 1997). The literature-identified peptides (Table 1) show that the length range is 15–50 with a mean length of 25 residues. Below the first interquartile of 19 are formaecin 1 and 2, apidaecin 1a/1b and metalnikowin 1, while above the third interquartile of 32 are Bac7(1–35), BnPRP1, BSN-37, PR-39, and penaeidin-1. Based on these literature-identified peptides, it appears that this “strict” length constraint (18–34 residues) does not accurately describe recognized members of the PrAMP family. Therefore, the length may simply provide an indication as to how “PrAMP-like” a given peptide is as many are within this “strict” length constraint. It is also important to remember that many of the PrAMPs have intracellular targets and peptide length/size is a significant factor in membrane permeability.

Again, considering the DRAMP database, there is a length range of 2–105 with just 1,784 sequences (37.6%) meeting the literature-provided “strict” length constraint of 18–34. While most cell penetrating peptides have been described as being 5–30 residues in length (Derakhshankhah and Jafari, 2018), the PrAMP bactenecin 7 is composed of 60 residues and is known to rapidly penetrate the bacterial membrane (Skerlavaj et al., 1990) to exert its effect intracellularly. Thus, we propose that length constraints are not necessary to either describe or omit peptides from the PrAMP family but can serve to indicate likely members.

### Proposed New Definition of a PrAMP

Based on the above analyses, we propose a more comprehensive set of criteria that define the PrAMP family. The proline content must make up at least 25% of the residues in the sequence. The peptides can be of any length, but they must be net cationic. Although peptides may exert their antimicrobial activity via multiple modes, it is essential that one be an intracellular target, i.e., inhibition of DnaK or the 70S ribosome. Given that naturally occurring AMPs are known to work synergistically (Gueguen et al., 2009), it may be that a poorly membrane-permeable peptide is a DnaK inhibitor though is reliant on other AMPs to disrupt the membrane initially. Conversely, our group has demonstrated that the PrAMP consensus sequence APO, a confirmed DnaK inhibitor, shows significant membrane disruption upon multimerization indicating that it may be both lytic and DnaK inhibiting in this multimer state (Li et al., 2015, 2016). This is an important factor as we assess known AMPs whose most common mode of action is lysis, then identifying PrAMP-like peptides from this set might mean there is the potential for peptides with a dual-mode of action (i.e., lytic and DnaK/70S ribosome inhibition). Furthermore, despite the fact that PRP motifs are common in PrAMPs and assist with destabilizing α-helical structures, activity at DnaK does not require the PRP motif, as is seen with DnaK-binding peptides NRLLLTG and ELPPVKI (Zahn et al., 2013). Instead, the PRP motif might simply provide an indication that the peptide has activity against DnaK.

Our newly proposed definition of members of the PrAMP family is summarized in Box 1. Under this definition, BnPRP1 (isolated from the plant *Brassica napus*) and the full-length penaeidin-1 (shrimp derived) are the only peptides in Table 1 which would be omitted from the PrAMP family due to an overall neutral net charge and significantly lower proline content (16% vs. 25% cut-off), respectively. In fact, despite being reported in a recent review as DnaK inhibitors (Mishra et al., 2018), we could find no literature precedent for this. Additionally, while BnPRP1 is proline-rich, it bears little similarity to members of the PrAMP family (Cao et al., 2015) as is the case for the penaeidin-1 (Destoumieux et al., 1997). This is further supported by the phylogenetic analysis in Figure 3 that shows both BnPRP1 and penaedin-1 clustering at the greatest distance from the rest of the family (i.e., furthest to the right). Interestingly, the PRD of penaeidin-1 does meet the proline and charge definitions (29% and +4) for a PrAMP and also contains the PRP motive. It is likely to be antimicrobial, as indicated by the antimicrobial activity of the PRD from the related penaedin4-1 (Cuthbertson et al., 2006) and, as such, is a promising replacement for the full-length penaedin-1 in the PrAMP family. Consequently, the PRD should have its antimicrobial activity confirmed with *in vitro* assays and then be tested for activity against DnaK.

Box 1 **Proposed new definitions of PrAMPs.**

**Table:** 

• **Proline Content:**	≥25% Proline
• **Antimicrobial:**	Essential
• **Intracellular Target:**	Essential (DnaK and/or 70S ribosome)
• **Key Motifs:**	PRP (indicative but not essential)
• **Net Charge:**	≥+1

## Expanding the PrAMP Family

Based on the new proposed definitions of PrAMPs (Box 1), we computationally interrogated the DRAMP database with the aim of identifying potential new members of the PrAMP family. We also included recently published peptides Tur1A/B (Mardirossian et al., 2018), to assemble an initial pool of 4,745 unique AMP sequences. Within the constraints of a minimum proline content of 25% and a minimum net charge of +1, a total of 75 (1.6%) “PrAMP-like” sequences were identified from the initial pool. Briefly, the new subset contained sequences with a length range of 6–79 residues, a proline content range of 25–53%, and a charge range of +1 to +20. Interestingly, the PRP motif occurred in 34 of the 75 sequences (45%) and often occurred multiple times in a single sequence, up to a maximum of 12 times (bactenecin 7). As outlined in the following sections, we considered a subset of these sequences that met the literature constraints for PrAMPs, or contained the PRP motif, as the most likely to represent new members of the PrAMP family.

### PrAMP-like Peptides Meeting Literature Constraints

To focus our investigation, we considered sequences that met the literature descriptions for PrAMPs, specifically sequences with >25% proline, 18–34 residues in length and that were cationic. A total of 32 sequences were identified that matched these constraints. As would be expected, several of the known PrAMPs fit these criteria and were selected including abaecin, apidaecin, Bac5(1–23), heliocin, drosocin, and others. Given these constraints, however, several commonly recognized PrAMPs are absent from the list, including Bac7(1–35), formaecin, metalnikowin, penaeidin-1, PR-39, and BnPRP1. This might indicate that these constraints are too strict to capture the “known” PrAMPs and supports our notion that an improved definition for PrAMPs is required. Nevertheless, it is interesting to observe which other AMPs (from the DRAMP database) fit these criteria. Specifically, the alpha-defensin-related sequences 7/10/12, antibacterial 6.5 kDa protein, antibacterial napin, lebocin-1/2 as well as P9 and PP30 all fit these criteria and are discussed below. Overall, there is nothing particularly remarkable about the newly identified sequences: they have proline content range of 25–38%; a length range of 19–34; and a net charge range of +2 to +7, but they may indeed qualify for membership within the PrAMP family given their feature similarity to known PrAMPs.

### PrAMP-like Peptides Containing the PRP Motif

Additionally, we sought to evaluate the likelihood of other PRP-containing AMPs as PrAMPs. From the obtained 75 “PrAMP-like” sequences in DRAMP database, we identified 34 sequences containing the PRP motif. Much like the earlier “strict” constraint analysis, a number of known PrAMPs are present, whilst a few remain absent, for example, heliocin and Bac5(1–23) do not contain the PRP motif and neither does abaecin from the honeybee *Apis mellifera*, but PRP is present in abaecin from the brown bumblebee *Bombus pascuorum*. Interestingly, a host of AMPs containing the PRP motif (not previously identified as PrAMPs) were identified including the antibacterial 6.5 kDa protein, astacidin 2, Cg-lgPrp and Cg-lgPrp P/Q, and PR-bombesin (explored in detail in section Putative New Members of the PrAMP Family).

### Putative New Members of the PrAMP Family

**Figure 4:**
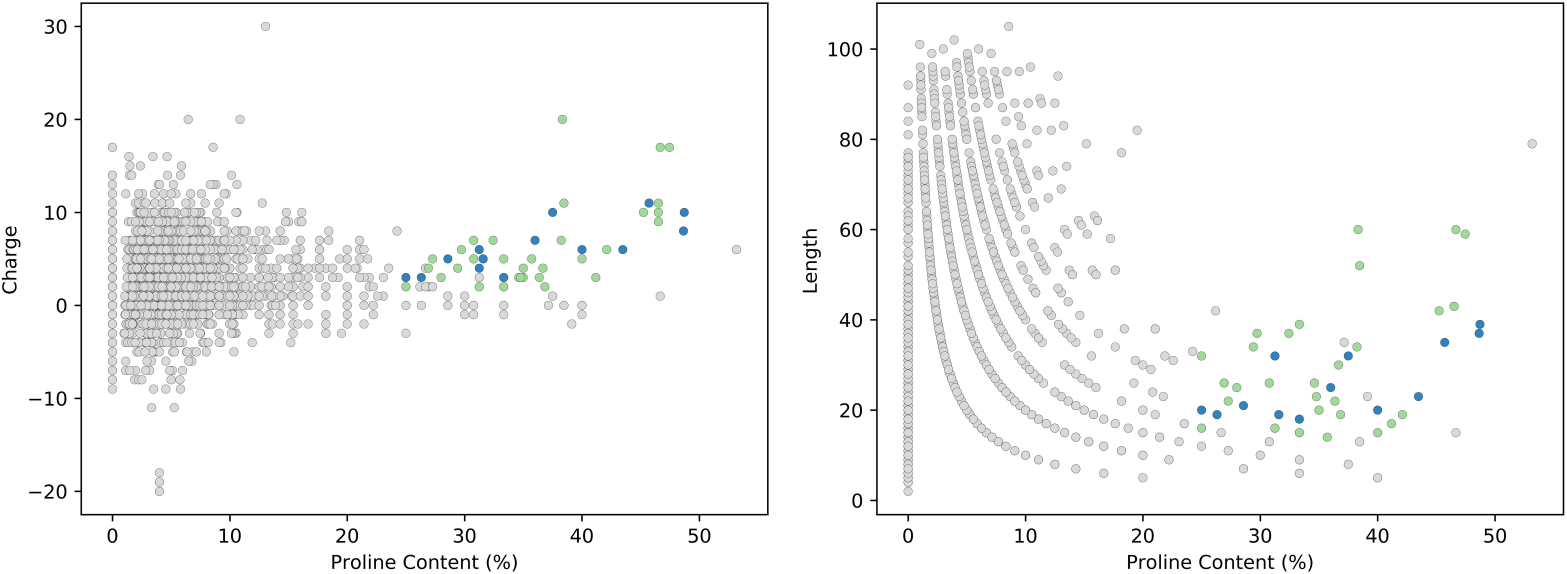
Analysis of the DRAMP database. PrAMP-like peptides that are putative new members of the PrAMP family are shown in green and currently reported PrAMP members are shown in blue. All other AMPs from the DRAMP database are shown in gray.

Our analysis of the DRAMP database revealed 75 sequences meeting the proposed criteria for new members of the PrAMP family as outlined in Box 1. From these sequences, we rationalized that the most likely new members of the PrAMP family would be those either meeting the literature constraints for a PrAMP, those containing the PRP motif, or both. This subset contained 55 peptides (shown in Table 2) and is represented graphically in Figure 4 where putative new members are shown in green and currently reported PrAMPs are shown in blue. All other peptides from the DRAMP database are shown in gray.

**Table 2:** PrAMP-like peptides most likely to be members of the PrAMP family.

**Name**	**Sequence**	**Length**	**Pro (%)**	**Net charge**	**PRP motifs**
Abaecin	YVPLPNVPQPGRRPFPTFPGQGPFNPKIKWPQ	32	31%	+4	0
Abaecin (*Bombus pascuorum*)	FVPYNP**PRP**GQSKPFPSFPGHGPFNPKIQWPYPLPNPGH	39	33%	+3	1
Abaecin (*Apis mellifera*)	YVPLPNVPQPGRRPFPTFPGQGPFNPKIKWPQGY	34	29%	+4	0
*Alpha-defensin-related sequence 10	PPCPSCPSCPWCPMCPRCPSCKCNPK	26	35%	+3	0
*Alpha-defensin-related sequence 12	PPCPSCLSCPWCPRCLRCPMCKCNPK	26	27%	+4	0
*Alpha-defensin-related sequence 7	PRCPPCPRCSWCPRCPTCPRCNCNPK	26	31%	+5	0
*Antibacterial 6.5 kDa protein	XXVPY**PRP**F**PRP**PIG**PRP**LPFPGGGRPFQS	30	37%	+4	3
*Antibacterial napin	PAQPFRFPKHPQGPQTRPPI	20	35%	+3	0
Apidaecin (Bombus pascuorum)	GNRPVYIPP**PRP**PHPRL	17	41%	+3	1
Apidaecin Cd3+	GKPSK**PRP**APIK**PRP**PHPRL	20	40%	+6	2
Apidaecin-1A	GNNRPVYIPQ**PRP**PHPRI	18	33%	+3	1
Apidaecin-1B	GNNRPVYIPQ**PRP**PHPRL	18	33%	+3	1
Apidaecin-2	GNNRPIYIPQ**PRP**PHPRL	18	33%	+3	1
APO	RPDK**PRP**YL**PRPRPPRP**VR	19	42%	+6	3^#^
Arasin 2	SRWPSPGR**PRP**FPGRPNPIFR**PRP**CICVRQPCPCDTY	37	30%	+6	2
Arasin1	SRWPSPGR**PRP**FPGRPKPIFR**PRP**C	25	36%	+7	2
Arasin-1	SRWPSPGR**PRP**FPGRPKPIFR**PRP**CNCYAPPCPCDRW	37	32%	+7	2
Astacidin 2	R**PRP**NYR**PRP**IYRP	14	36%	+5	2
*Attacin-C	QRPYTQPLIYYPPPPTPPRIYRA	23	35%	+3	0
Bac5(1–23)	RFRPPIRRPPIRPPFYPPFRPPI	23	43%	+6	0
Bac7(1–35)	RRIR**PRP**PRL**PRPRPRP**LPF**PRP**G**PRP**I**PRP**LPFP	35	46%	+11	6^#^
Bactenecin 5	RFRPPIRRPPIRPPFYPPFRPPIRPPIFPPIRPPFRPPLRFP	42	45%	+10	0
Bactenecin 7	RRIR**PRP**PRL**PRPRPRP**LPF**PRP**G**PRP**I**PRP**LPF**PRP**G**PRP**I**PRP**LPF **PRP**G**PRP**I**PRP**L	60	47%	+17	12^#^
BSN-37	FRPPIRRPPIRPPFYPPFRPPIRPPIFPPIRPPFRPP	37	49%	+8	0
Cathelicidin-2 (*Bos taurus*)	RFRPPIRRPPIRPPFYPPFRPPIRPPIFPPIRPPFRPPLGPFP	43	47%	+9	0
Cathelicidin-2 (*Capra hircus*)	RFRPPIRRPPIRPPFNPPFRPPVRPPFRPPFRPPFRPPIGPFP	43	47%	+10	0
Cathelicidin-2 (*Ovis aries*)	RFRPPIRRPPIRPPFRPPFRPPVRPPIRPPFRPPFRPPIGPFP	43	47%	+11	0
Cathelicidin-3 (*Bos taurus*)	RRIR**PRP**PRL**PRPRPRP**LPF**PRP**G**PRP**I**PRP**LPF **PRP**G**PRP**I**PRP**LPF**PRP**G**PRP**I**PRP**	59	47%	+17	12^#^
Cathelicidin-3 (*Ovis aries*)	RRLRPRRPRL**PRPRPRPRPRP**RSLPL**PRP**QPRRI**PRP**ILLPWRP**PRP** I**PRP**QPQPIPRWL	60	38%	+20	7^#^
Cathelicidin-3.4 (*Capra hircus*)	RFRLPFRRPPIRIHPPPFYPPFRRFL	26	31%	+7	0
Cg-lgPrp	GPIRRPK**PRPRPRP**E	15	40%	+5	2^#^
Cg-lgPrp P/Q	GPIRRPK**PRP**RQRPE	15	33%	+5	1
Dros pro attC	RPYTQPLIYYPPPPTPPRIYRA	22	36%	+3	0
Drosocin	GK**PRP**YS**PRP**TSH**PRP**IRV	19	32%	+5	3
Dros-Pyrr-Dros	GK**PRP**YL**PRP**TSH**PRP**IRV	19	32%	+5	3
Heliocin	QRFIHPTYRPPPQPRRPVIMRA	22	27%	+5	0
Heliocin	RFIHPTYRPPPQPRRPVIMRA	21	29%	+5	0
Lebocin-1/2	DLRFLYPRGKLPVPTPPPFNPKPIYIDMGNRY	32	25%	+3	0
Metalnikowin-1	VDKPDYR**PRPRP**PNM	15	33%	+2	1^#^
Metalnikowin-2A	VDKPDYR**PRP**W**PRP**N	15	33%	+2	2
Metalnikowin-2B	VDKPDYR**PRP**WPRNMI	16	25%	+2	1
Metalnikowin-3	VDKPDYR**PRP**W**PRP**NM	16	31%	+2	2
Metchnikowin	HRHQGPIFDTRPSPFNPNQ**PRP**GPIY	26	27%	+2	1
Metchnikowin-2	RRQGPIFDTRPSPFNPNQ**PRP**GPIY	25	28%	+3	1
OaBac6	RRLRPRHQHFPSERPWPKPLPLPL**PRP**G**PRP**WPKPLPLPL**PRP**GLRPWPKPL	52	38%	+11	3
Oncocin	VDKPPYL**PRPRP**PRRIYNR	19	32%	+5	1^#^
Oncopeltus antibacterial peptide-4	VDKPPYL**PRP**PPPRRIYNNR	20	35%	+4	1
*P9	RFIPPILRPPVRPPFRPPFRPPFRPPPIIRFFGG	34	38%	+7	0
*PP30	YVPPVQKPHPNGPKFPTFP	19	37%	+2	0
PR-39 (Antibacterial protein PR-39)	RRR**PRP**PYL**PRPRP**PPFFPPRLPPRIPPGFPPRFPPRFP	39	49%	+10	2^#^
PR-bombesin	EKKP**PRP**PQWAVGHFM	16	25%	+2	1
Pyrrhocoricin	VDKGSYL**PRP**TP**PRP**IYNRN	20	25%	+3	2
Riptocin	VDKGGYL**PRP**TP**PRP**VYRS	19	26%	+3	2
Tur1A	RRIRFRPPYL**PRP**GRRPRFPPPFPIPRIPRIP	32	38%	+10	1
Tur1B	RRIPFWPPNWPGPWLPPWSPPDFRIPRILRKR	32	31%	+6	0

Putative new members are marked with an asterisk (*). The hash (^#^) indicates overlapping PRP motifs. PRP motifs are bolded.

Interestingly, applying these “strict” constraints to the current PrAMP members in Table 1, BnPRP1 is omitted as described earlier. Additionally, whilst containing sufficient proline content, the lack of a PRP motif and containing just 16 and 15 residues, respectively, formaecin 1 and 2 would also be omitted. The formaecins (GRPNPVNN/TKPTPY/HPH/RL) share reasonable similarity to droscocin (GKPRPYSPRPTSHPRPIRV) but the PYS and PIR motifs responsible for forward and reverse DnaK binding in drosocoin (Graf and Wilson, 2019), respectively, are absent. In the case of PYS, this is substituted for PVN in the formaecins but the PVN motif has not been reported as DnaK binding nor is present in any other PrAMP in Table 2. To our knowledge, formaecin has not been reported to inhibit DnaK or the 70S ribosome. Although the formaecins are indeed very similar to many PrAMPs, they too would need to be confirmed as DnaK or 70S ribosome inhibitors before (re)admission to the PrAMP family and, upon doing so, would expand the understanding of recognized motifs responsible for DnaK or 70S ribosome binding.

From our analysis, we assessed putative new PrAMP members from Table 2. A small number of these peptides (astacidin, Cg-PRP, lebocins, and PR-bombesin) appear in an early review regarding PrAMPs (Scocchi et al., 2011). However, those marked with asterisk (^*^) appear to be not reported as PrAMPs in that review but are also likely PrAMPs according to our analysis. In the following sections, we will discuss each of the peptides or peptide families as potential members of the PrAMP family and propose their likelihood of membership.

#### Alpha-defensin-related Sequences (ADRS) 7, 10, and 12

The ADRS 7, 10, and 12 (ADRS7: PRCPPCPRCSWCPRCPTCPRCNCNPK) are also known as cryptidin-related sequences and were discovered in mouse Paneth cells as cysteine-rich and proline-rich peptides containing the C-P-Xaa repeat (Huttner and Ouellette, 1994; Hornef et al., 2004). While a mechanism of action has not been proposed specifically for ADRS-7,10,12, ADRS-2 (also known as CRS4C-1) has been shown to be membrane disruptive and to permeabilize *E. coli* (Shanahan et al., 2010). This peptide family also demonstrated that it can form homodimers that increase bactericidal activity (Hornef et al., 2004). Whilst the ADRS-7,10,12 peptides match the literature description for the PrAMPs and are indeed proline-rich, they may not necessarily behave like PrAMPs. That is to say that they are likely to be only membrane-disruptive and without intracellular targets. The presence of 3 intramolecular disulfide bonds also has a high level of secondary structure that differs from the flexible non-structured peptides in the PrAMP family. Overall, the ADRSs cannot be considered members of the PrAMP family.

#### Antibacterial 6.5 kDa Protein

The antibacterial 6.5 kDa protein (XXVPYPRPFPRPPIGPRPLPFPGGGRPFQS) is proline-rich and was identified from the haemocytes of the shore crab (Schnapp et al., 1996). It shares >60% sequence similarity with bactenecin 7 including the 3 repeats of the PRP motif. Bactenecin 7 is a known inhibitor of protein synthesis by targeting 70S ribosomes and inhibits DnaK. It is unknown if this peptide is a DnaK binder but given its similarity to bactenecin 7, and reasonable similarity to another crab peptide, arasin-1 (SRWPSPGRPRPFPGRPKPIFRPRPCNCYAPPCPCDRW), a known DnaK inhibitor (Stensvag et al., 2008), it is highly likely that the antibacterial 6.5 kDa protein is a DnaK inhibitor and thus should be considered a member of the PrAMP family together with an updated name.

#### Antibacterial Napin (Napin-like Polypeptide)

The antibacterial napin sequence reported in the DRAMP database (DRAMP03467: PAQPFRFPKHPQGPQTRPPI) is reported as a napin-like polypeptide in the literature (Ngai and Ng, 2004) where the first 10 residues at N-terminus share a high sequence similarity to the AQPFRFKKTEXTTT sequence from the Cathelicidin antimicrobial peptides (*Mus musculus*) and the cathelin-related antimicrobial peptide (*Mus musculus*) (Ngai and Ng, 2004). The C-terminal 10 residues share a high sequence similarity with PQGPQQRPPTEXTTT found in antifungal 2S albumin large chain (*Raphanus sativus*) and trypsin inhibitor (*Sinapis arvensis*) (Ngai and Ng, 2004). The napin-like polypeptide indeed shares these properties exhibiting inhibition of trypsin, inhibition of cell-free translation in rabbit reticulocyte lysate, and inhibition of bacterial growth in *Pseudomonas fluorescens, Mycobacterium phlei, Bacillus cereus, Bacillus subtilis*, and *Bacillus megaterium* but not *Proteus vulgaris, Staphylococcus aureus, E. coli, Enterobacter aerogenes* nor *Pseudomonas aeruginosa*. Despite not containing the PRP motif specifically, our analysis suggests that this napin-like polypeptide could also be a DnaK binder and should be considered a member of the PrAMP family.

#### Astacidin 2

Astacidin 2 (RPRPNYRPRPIYRP) was discovered in hemocytes from freshwater crayfish and has activity against both Gram-positive and Gram-negative bacteria (Jiravanichpaisal et al., 2007). Astacidin not only contains the PRP motif but also specifically the PRPIY motif implicated in the reverse binding mode of pyrrhocoricin to DnaK (Zahn et al., 2013). Despite this fact, astacidin 2 appears not to have been proposed as a potential DnaK binder. Our analyses suggest that astacidin 2 is very likely a DnaK binder and, correspondingly, a likely member of the PrAMP family.

#### Attacin-C

Attacin-C (QRPYTQPLIYYPPPPTPPRIYRA)was isolated from the *Drosophila melanogaster* fruit fly and shares remarkable similarity to many of the known PrAMPs, particularly pyrrhocoricin. Interestingly, attacin-C exhibited effectively no inhibition of growth against multiple strains of Gram-positive and Gram-negative bacteria, yeast and fungi, leading the authors to demonstrate that, instead, it has synergistic antibacterial activity with cecropin A (an antimicrobial peptide known to improve membrane permeability) (Rabel et al., 2004). While its intracellular target and mode of action have yet to be identified, (Rabel et al., 2004), given the similarity to known PrAMPs and its synergistic activity, attacin-C may be a DnaK inhibitor despite not appearing to be membrane permeable. At this point, without confirmed antimicrobial activity, attacin-C may be best considered as an AMP adjuvant (Sheard et al., 2019).

#### Cg-lgPrp, Cg-lgPrp P/Q (and Cg-Prp)

Cg-lgPrp (GPIRRPKPRPRPRPE) and Cg-lgPrp P/Q (GPIRRPKPRPRQRPE) are truncated (long: lg) synthetic variants derived from the proline-rich peptide Cg-Prp (ILENLLARSTNEDREGSIFDTGPIRRPKPRPRPRPEG) identified from oysters (Gueguen et al., 2009; Schmitt et al., 2012a). They each have at least one PRP motif and have strong sequence similarity with other PrAMPs particularly metalnikowin-1, a known DnaK inhibitor. Despite the speculation that Cg-Prp might have an intracellular target (Schmitt et al., 2012b) it appears not to have been proposed as a DnaK inhibitor. This family is very likely to comprise DnaK binders and should be given a membership within the PrAMP family. However, the full-length sequence falls outside of the 25% proline constraint containing just 16% proline and the C-terminus half of the peptide shares little similarity with PrAMPs.

#### Lebocin-1/2

Lebocin-1/2 (DLRFLYPRGKLPVPTPPPFNPKPIYIDMGNRY) was isolated from the *Bombyx mori* silk moth and unsurprisingly, belongs to the lebocin family (Hara and Yamakawa, 1995). Interestingly, heliocin (QRFIHPTYRPPPQPRRPVIMRA), a recognized PrAMP, is also a member of the lebocin family and a known reverse binder of DnaK through the PVI motif (Zahn et al., 2013). While lebocin-1/2 lacks the PVI motif specifically, it shares similarity to pyrrhocoricin (and attacin-C) in the PTPP motif where these and other proline residues have been suggested to contribute to structural stability of the pyrrhocoricin (Bower et al., 2003). Fragments of lebocin 1 have recently been reported to disrupt cell membranes of *E. coli* and yet, for *S. aureus*, it inhibited cell division with minimum damage to the surface (Yang et al., 2020). These data and these results found here suggest that lebocin-1/2 represents a probable member of the PrAMP family.

#### P9 (a Deer Cathelicidin)

The P9 peptide (RFIPPILRPPVRPPFRPPFRPPFRPPPIIRFFGG) was identified from New Zealand deer blood with striking similarity to the proline/arginine-rich cathelicidins, such as Bac5(1–23), from other mammals containing the Xaa-R-P-P repeat motif. Bac5(1–23) is a PrAMP and known DnaK inhibitor also identified from neutrophils. It is highly likely that the deer P9 peptide is also a DnaK inhibitor and thus a member of the PrAMP family.

#### PP30 (a Wasp Abaecin-like Peptide)

The PP30 peptide (YVPPVQKPHPNGPKFPTFP) was identified from wasps and has a high sequence similarity to the abaecin precursor (UniProt: P15450: YVPLPNVPQPGRRPFPTFPGQGPFNPKIKWPQGY) (Shen et al., 2010). Interestingly, while the PP30 peptide and the abaecin precursor are both antibacterial, they lack the WPYPLPN motif, predicted by computational modeling to be responsible for DnaK inhibition (Rahnamaeian et al., 2015), that is present in Abaecin (UniProt: P81463: FVPYNPPRPYQSKPFPSFPGHGPFNPKIQWPYPLPNPGH). This prediction has yet to be confirmed, nor has PP30 been assessed for DnaK inhibition. Excitingly, as PP30 exhibits membrane-rupturing properties uncommon of PrAMPs (Shen et al., 2010) if it is also active at DnaK or the 70S ribosome, then it would represent a novel dual-mode of action addition to the PrAMP family.

#### PR-bombesin

PR-bombesin (EKKPPRPPQWAVGHFM) was isolated from the toad *Bombina maxima* and unlike other bombesin-related peptides, it is the only proline-rich peptide, hence the terminology PR (Lai et al., 2002). PR-bombesin has antimicrobial activity against *E. coli* and *S. aureus*, an effect that is completely abolished by the substitution of the proline residues to glycine (Li et al., 2006). The presence of the PRP motif indicates that, like other PrAMPs, it may have DnaK inhibitory effects despite the fact that PrAMPs have rarely been reported in frogs. PR-bombesin is likely a DnaK binder and would therefore, be a member of the PrAMP family.

## Conclusions

PrAMPs are a fascinating class of membrane permeable peptides with intracellular targets of DnaK or the 70S ribosome. Based on a rigorous analysis of known PrAMPs from the literature, we propose a new set of definitions to describe members of the PrAMP family. Under these definitions, we examined AMPs from the DRAMP database to identify peptides with PrAMP-like properties. We assessed these PrAMP-like peptides in two ways: firstly, to identify peptides matching the “strict” definitions of PrAMPs from the literature, and secondly, to identify peptides containing the commonly recognized PRP motif. From these approaches we identified an additional 10 (single peptides or peptide families) from diverse species, that are most likely to be new members of the PrAMP family. Detailed literature assessment suggests that all of these families, bar ADRSs and potentially attacin-C, are likely to be members of the PrAMP family subject to subsequent demonstration of DnaK or 70S ribosome inhibition studies. Overall, this body of analysis provides a new definition for PrAMPs and provides a list of putative members for the discovery of novel PrAMP candidates.

## Methods

### DRAMP Database

The DRAMP database (hosted at http://dramp.cpu-bioinfor.org/) (Kang et al., 2019) was accessed in June 2020 and the DRAMP_Antimicrobial_amps.xlsx file was used for analysis. The dataset was consolidated with PrAMPs identified from the literature and duplicate entries were removed.

### Evolutionary Relationships of Taxa

The evolutionary history of PrAMPs was inferred using the Neighbor-Joining method (Saitou and Nei, 1987). The optimal tree with the sum of branch length = 7.43427792 is shown. The tree is drawn to scale, with branch lengths in the same units as those of the evolutionary distances used to infer the phylogenetic tree. The evolutionary distances were computed using the Poisson correction method (Zuckerkandl and Pauling, 1965) and are in the units of the number of amino acid substitutions per site. This analysis involved 21 amino acid sequences. All ambiguous positions were removed for each sequence pair (pairwise deletion option). There were a total of 63 positions in the final dataset. Evolutionary analyses were conducted in MEGA X (Kumar et al., 2018).

## Data Availability Statement

The datasets presented in this study can be found in online repositories. The names of the repository/repositories and accession number(s) can be found in the article/supplementary material.

## Author Contributions

All authors listed have made a significant intellectual and written contribution to the work and have approved it for publication.

## Conflict of Interest

The authors declare that the research was conducted in the absence of any commercial or financial relationships that could be construed as a potential conflict of interest.
